# Prevention of dendritic and synaptic deficits and cognitive impairment with a neurotrophic compound

**DOI:** 10.1186/s13195-017-0273-7

**Published:** 2017-06-27

**Authors:** Narjes Baazaoui, Khalid Iqbal

**Affiliations:** 1 Department of Neurochemistry, IngeGrundke-Iqbal Research Floor, New York State Institute for Basic Research in Developmental Disabilities, 1050 Forest Hill Road, Staten Island, NY 10314 USA; 2 Graduate Program in Biology (Neuroscience), College of Staten Island (CSI), City University of New York (CUNY) Graduate Center, 365 Fifth Avenue, New York, NY 10016-4309 USA

**Keywords:** Alzheimer’s disease, 3 × Tg-AD mice, Synaptic plasticity, Cognitive deficit, Synaptic compensation, Neurotrophic compound, Neurogenesis, Prevention

## Abstract

**Background:**

The use of neurotrophic factors to treat Alzheimer’s disease (AD) is hindered by their blood–brain barrier impermeability, short half-life, and severe side effects. Peptide 021 (P021) is a neurotrophic/neurogenic tetra-peptide that was derived from the most active region of the ciliary neurotrophic factor (CNTF) by epitope mapping. Admantylated glycine was added to its C-terminal to increase its blood–brain barrier permeability and decrease its degradation by exopeptidases to make it druggable. Here, we report on the preventive effect of P021 in 3 × Tg-AD, a transgenic mouse model of AD.

**Methods:**

P021 was administered in the diet at 3 months, i.e., 6–9 months before any overt amyloid beta (Aβ) or tau pathology, and during the period of synaptic compensation, and was continued until 21 months in 3 × Tg-AD mice. The 3 × Tg-AD mice and wild-type (WT) mice were treated identically but with a vehicle-only diet serving as controls. The effects of P021 on neurogenesis, dendritic and synaptic markers, and cognitive performance were investigated.

**Results:**

We found that P021 treatment was able to rescue dendritic and synaptic deficits, boost neurogenesis, and reverse cognitive impairment in 3 × Tg-AD mice.

**Conclusions:**

Availability of appropriate neurotrophic support during the period of synaptic compensation can prevent synaptic deficit and cognitive impairment, and P021 is a promising neurotrophic compound for this purpose.

**Electronic supplementary material:**

The online version of this article (doi:10.1186/s13195-017-0273-7) contains supplementary material, which is available to authorized users.

## Background

Alzheimer’s disease (AD) is characterized histopathologically by an extensive neurodegeneration, especially of the hippocampus, that is associated with intraneuronal neurofibrillary tangles of abnormally hyperphosphorylated tau and extracellular deposition of amyloid beta (Aβ) as plaques. Neurodegeneration that results in dementia is seen both as neuronal and synaptic loss. During the last two decades, the vast majority of the therapeutic approaches have focused mainly on inhibiting neurodegeneration by prevention or clearance of Aβ plaques. However, so far results have been disappointing and, to date, no disease-modifying drug has been approved for the treatment of AD. These repeated failures could be due to either inhibition of Aβ alone not being sufficient, and/or that the treatment was initiated too late to be effective.

During the disease process, the AD brain tries to compensate for synaptic and neuronal loss by initiating the dentate gyrus (DG) neurogenesis and dendritic sprouting but, because of insufficient appropriate neurotrophic support, this attempt does not succeed in replacing neuronal loss with new mature functional neurons nor in replacing the lost dendrites and synapses [1–7].

A key mechanism of neuronal loss and neurogenesis and synaptic plasticity deficits could be the imbalance of neurotrophic factors [7]. Indeed, it is known that the levels of fibroblast growth factor (FGF)2 are increased and that of brain-derived neurotrophic factor (BDNF) and neurotrophin-4 are decreased in the AD brain [8–10]. Thus, one therapeutic approach is to provide the diseased brain with neurotrophic support especially during the period of synaptic compensation to help the self-repair attempt of the brain.

Previously, we showed that ciliary neurotrophic factor (CNTF) can counteract the mitogenic effect of FGF2 [11] and, based on this finding, we developed a CNTF peptidergic compound, P021, which can increase the transcription of BDNF, enhance neurogenesis and neuronal plasticity, and improve cognitive performance in mice and rats [12, 13]. Here, we investigated the beneficial therapeutic effect of boosting the neurotrophic environment by initiating treatment with P021 in the diet at 3 months of age (i.e., around 6–9 months before any overt Aβ or tau pathology, and during the period of synaptic compensation) and continuing for approximately 18 months in a triple transgenic mouse model of AD (3 × Tg-AD mice). This mouse model that overexpresses a human βAPP_Swe_ and tau P301L in PS1_M146v_ knock-in background, although a genetic mixture of AD and fronto-temporal dementia mutations, shows synaptic compensation associated with cognitive impairment at the age of 12–16 weeks, and Aβ plaque and tau pathologies several months later [14, 15]. The initiation of treatment with P021 during the period of synaptic compensation rescued neurogenesis and synaptic deficits and reversed cognitive impairment at around 9 and 15–16 months posttreatment in 3 × Tg-AD mice.

## Methods

### Animals and housing

Animals were housed and bred following our Institutional Animal Care and Use Committee (IACUC) guidelines, according to the PHS Policy on Human Care and Use of Laboratory Animals (revised January 2013). Animals were group-housed at five animals per cage with a standard temperature of 22 ± 1°C and a light-controlled environment (12:12 h light/dark cycle), and with ad libitum access to food and water. Breeding pairs of the 3 × Tg-AD mice expressing three human mutation transgenes (APP_Swe_ mutation, tau P301L mutation, and Presenilin1 (PS1) knock-in) and wild-type (WT) age-matched control mice from the same genetic background (hybrid 129/Sv × C57BL/6) were obtained from Dr. Frank LaFerla through the Jackson laboratory (New Harbor, ME, USA). In this mouse model the extracellular deposition of the Aβ plaques starts at around 9 months in the neocortex and expands to the hippocampus at around 12 months, and the formation of neurofibrillary tangles (NFT) starts at around 12 months in the limbic areas and expands to the other brain regions several months later. Furthermore, Aβ and tau pathologies develop in a progressive age-dependent manner with similar brain distribution of the lesions, as in AD. However, 3 × Tg-AD mice are homozygous and were independently bred from 129/Sv × C57BL/6 WT mice, and thus the latter were not ideal littermate controls to the transgenic mice. A period of acclimatization from 45 min to 1 h was accorded to the animals before each behavioral test to habituate to the new environment. Only female 3 × Tg-AD and age-matched WT control mice were used in the study because, similar to humans, female 3 × Tg-AD mice show more aggressive pathology with earlier cognitive impairment, more Aβ deposition, and a higher and earlier deficit in neurogenesis than their male counterparts [16–18]; the female transgenic mice show overt Aβ and tau pathologies at around 9 and 12 months, respectively [14]. While originally both male and female 3 × Tg-AD mice were reported by Dr. La Ferla to show AD pathology, the subsequent studies found that only female 3 × Tg-AD animals show more consistent and aggressive pathology [16–19]. Indeed, they are known to exhibit more aggressive Aβ pathology compared to males during the plaque-bearing stage, and Aβ pathology becomes more evident at 14 months in females compared to males. Furthermore, at 16 months of age female mice exhibit higher levels of soluble Aβ1–40 and 1–42 as well as total Aβ levels, and the difference becomes clearer at 23 months. Female mice have higher Aβ secretase activity and less efficient Aβ degradation than males, and there is no difference in tau pathology [17, 18]. To have consistent and reliable data that can be replicated we thus decided to use female mice instead of using both sexes. Animals other than the ones employed for behavioral studies were employed for immunohistochemical and biochemical studies to exclude any effect of behavioral studies on neurogenesis and synaptic plasticity (Additional file 1: Table S1).

### Study design

**Fig. 1:**
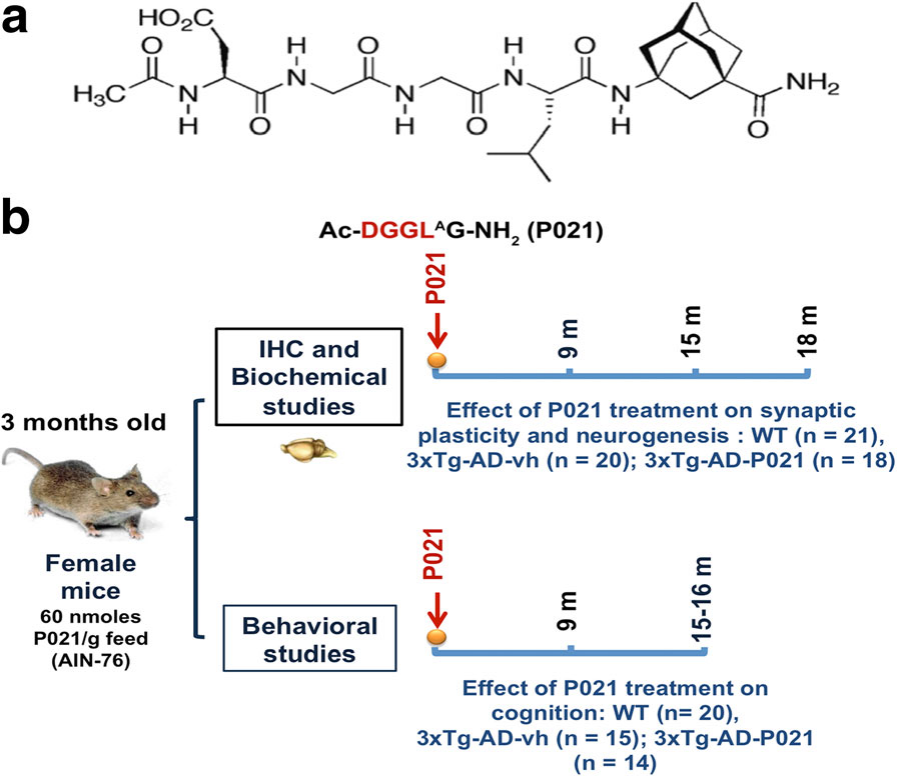
Chemical structure of P021 and study design. Female 3 × Tg-AD 3-month-old mice were put on mouse chow (AIN-76, Research Diets, New Brunswick, NJ, USA) that contained 60 nmol P021/g feed. As controls, both 3 × Tg-AD mice and gender-, age-, and genetic background-matched WT mice (129/Sv × C57BL/6) were treated with the vehicle diet. On average the mice consumed ~2.7 g diet/day which amounted to 60 nmol × 2.7 = 162 nmol P021 per mouse per day. At 9, 15, and 18 months posttreatment, mice were sacrificed and their brains studied for expressions and levels of various proteins associated with cognition both immunohistochemically and biochemically (see Figs. 2, 3, 4, 5, 6 and 7). Separate sets of animals were treated as above with a P021 or vehicle diet and tested behaviorally at 9 and 15–16 month posttreatment (Figs. 8 and 9). **a** The chemical structure of P021. **b** Study design. *3 × Tg-AD-P021* 3 × Tg-AD mice treated with a P021 diet, 3 × Tg-AD-vh 3 × Tg-AD mice treated with a vehicle diet, *IHC* immunohistochemical staining, *WT* wild-type control mice

Three-month-old female WT animals (*n* = 41), 3 × Tg-AD mice treated with a vehicle diet (3 × Tg-AD-vh; *n* = 35), and 3 × Tg-AD mice treated with a P021 diet (3 × Tg-AD-P021; *n* = 32) were used in the study. The animals were divided into two sets: one set of WT (*n* = 20), 3 × Tg-AD-vh (*n* = 15), and 3 × Tg-AD-P021 (*n* = 14) animals were used for behavioral studies, and another set of mice that did not go through behavioral studies (WT (*n* = 21), 3 × Tg-AD-vh (*n* = 20), and 3 × Tg-AD-P021 (*n* = 18)) were employed for immunohistochemical and biochemical analyses. The mice that were employed for behavioral analyses were used to study the effect of P021 on cognition. At 9 months after treatment with P021 the Morris Water Maze task was conducted. Then, at 15–16 months after treatment, the mice were tested by the object location task. Brain samples from mice that were not tested behaviorally were taken at the age of 3 months just before the start of the treatment as a baseline measure and then at 9, 15, and 18 months after treatment with P021 (Fig. 1b). P021 was given in the food diet at a concentration of 60 nmol/g feed.

### P021 treatment

At approximately 3 months of age, the animals were divided into three groups: one group of 3 × Tg-AD-P021 (*n* = 32) treated with 60 nmol P021/g feed in the diet; a second group of 3 × Tg-AD-vh (*n* = 35) treated with the same diet but without P021; and a third group of WT (*n* = 41) treated with the same diet but without P021. P021 was formulated by Research Diets (New Brunswick, NJ, USA). Food consumption was recorded every 2 weeks and body weight every month. The average mouse food consumption was ~2.7 g feed/day.

### Design and synthesis of P021

The chemical structure of P021 is shown in Fig. 1a. It is a pentapeptide (Ac-DGGL^A^G-NH2; molecular weight 578.3) generated by the addition of adamantylated glycine at the C-terminus of CNTF tetrapeptide (amino acid residues 148–151). The adamantylated glycine was added to P021 to increase its blood–brain barrier permeability and decrease its degradation by carboxy peptidases [8, 13, 20]. P021 was synthesized and purified by reverse phase HPLC to >96% purity as described previously [13]. The sequence of P021 was confirmed by mass spectrometry.

### P021 stability in plasma, gastric and intestinal juices, and blood–brain barrier permeability

P021 has >95% stability in artificial gastric juice over 30 min, ~100% stability in intestinal fluid over 120 min, a plasma stability >3 h, and is blood–brain barrier permeable [21].

### Behavioral studies

At 9 months posttreatment mice were tested for spatial reference memory by the Morris Water Maze task [22] and at 15–16 months posttreatment by a novel object location task.

#### Morris Water Maze task

The Morris Water Maze task was performed according to a procedure adapted from that described by Morris et al. [22]. Briefly, animals were divided into three groups: 3 × Tg-AD-vh (*n* = 15), 3 × Tg-AD-P021 (*n* = 14), and age-matched WT control mice (*n* = 20). A circular pool tank 180 cm in diameter was used and a 13-cm escape platform was submerged 1 cm below the surface of water in the Northwest quadrant. The escape platform was invisible to mice since the water was made opaque by a white nontoxic chalk. During each trial each animal was trained starting from a different quadrant so that it learns to associate the spatial cues to the place of the submerged platform. The water was kept at a constant temperature of 21 ± 1°C. Animals were trained for 4 consecutive days, with four trials per day. During each trial each animal was given 90 s to find the escape platform otherwise it was gently guided to it. The first probe trial (PT1) was given 24 h after the last training session and the second probe trial (PT2) was given 20 days after the first probe trial. Each animal was given 60 s to look for the previous place of the platform. Recording of the behavior of each mouse was performed using a Samsung digital camera (SDC 4304) mounted on the ceiling; SMART version 2.0.14 software (Pan Lab/San Diego Instruments) was used for tracking and timing of each trial.

#### Object location task

The object location task in the open field is a test for short-term spatial memory, which is mainly hippocampal-dependent. In this test, the 18-month-old animals were required to identify the location of the object as novel or familiar based on a previous experience. The test consists of a habituation phase, a sample phase, and a test phase. During the habituation phase animals underwent a total of six sessions, two sessions/day, and each session lasted 10 min within 2 h of intertrial interval. Neophobia measurements, in which an object was put in the center of the arena and the amount of exploration time was recorded, were performed during the first habituation phase. Twenty-four hours after the last habituation session the sample phase and the test phase of the novel object location were conducted. During the sample phase two identical objects were placed symmetrically in two different places, each 15 cm away from the corner, and animals were allowed to explore them for 5 min. One hour later, in the test phase, one of the two objects was moved to a new location 15 cm away from the corner, in a symmetrical position to the familiar one, and the time spent exploring each object was recorded. To evaluate cognitive performance a discrimination index was calculated as follows: ((time exploring the new location – time exploring familiar location)/total time exploring both locations) × 100. Mice were considered to be impaired if they spent more time investigating the familiar location than the novel one. After each trial the objects were removed and cleaned with 70% alcohol to remove any olfactory cues.

### Tissue processing

For immunohistochemical and biochemical analysis the animals were anesthetized using an overdose of avertin and then transcardially perfused using 0.1 M phosphate-buffered saline (PBS). The left hemisphere was dissected into the hippocampus and cortex (forebrain) and was employed for biochemical analysis. The right hemisphere was immersion-fixed with 4% paraformaldehyde in 0.1 M PBS for 24–48 h at 4°C then cryoprotected with 30% sucrose in 0.1 M PBS for 48–72 h at 4°C. Sagittal sections of 40 μm thickness were cut using a freezing-sliding microtome and were stored in glycol antifreeze solution at –20°C until used for immunohistochemical analysis.

### Immunohistochemistry

For immunohistochemistry four to five animals per group were chosen randomly. Every fifth section, five to six sections per mouse, were used for intensity-scanning analysis. For immunofluorescence, free-floating sections recovered from the glycol antifreeze solution were washed three times in 1× PBS for 15 min and then incubated in 0.5% Triton-X100 in PBS for 15 min. Sections were then washed three times in 1× PBS for 15 min each and blocked in 5% normal goat serum containing 0.05% Tween-20 and 0.05% Triton X-100 for 45 min. Sections were then incubated overnight at 4°C with the corresponding primary antibodies (see Additional file 1: Table S2 for all the antibodies that were used in this study). Treatment with the primary antibody was followed by treatment with the corresponding secondary fluorescent antibody, either Alexa 488-conjugated goat antimouse IgG antibody (1:500; Molecular Probes, Carlsbad, CA, USA) or CY3-conjugated goat antirabbit antibody (1:500; Jackson Laboratory, Bar Harbor, Maine, USA) for 2 h at room temperature protected from light. Sections were washed three times with 1× PBS for 20 min each and then mounted on microscope slides with fluorogel mounting medium (Electron microscopy, Hatfield, PA, USA). All the slides were then covered with coverslips and stored at 4°C until analyzed by confocal microscopy under identical conditions. For all the staining, maximum projection images were generated using the average of 15 Z-stacks using the 20× objective of a Nikon 90i fluorescent microscope equipped with a Nikon C1 three-laser confocal system and a Nikon DS U1 digital camera. The entire area of the DG, CA1 (stratum radiatum and stratum pyramidae), CA3 (stratum oriens, stratum radiatum, and stratum pyramidae), and parietal association cortex (all six layers) were analyzed. For each section the micrographs were transformed to grayscale using the imageJ software and then each picture was thresholded so that all the staining in the section with the background was quantified. Mean pixel intensity was measured for each brain region using the Image J software package (NIH). The area to be analyzed was selected and converted to greyscale then the threshold was adjusted and the mean pixel intensity of the region of interest was quantified.

To study neurogenesis, doublecortin (DCX) and Ki67 double immunohistochemical staining was performed. The same procedure as stated above was used for the immunohistochemical staining with the addition of the extra step of antigen retrieval. Sections were then counterstained with the nuclear staining TO-PRO-3 iodide (1:1000; Invitrogen, Camarillo, CA, USA) for 30 min and then washed two times for 20 min. Five mice per group and five sections (every tenth section) per brain per mouse were analyzed. All sections were carefully investigated for DCX-positive and the Ki67-positive staining. Labeled cells in the granule cell layer and the hilus of the DG of the hippocampus were manually quantified using a 40× oil objective of a Nikon 90i fluorescent microscope. The number of DCX-positive staining cells and Ki-67-positive cells was reported as the percent of control.

### Western blots

The brain tissue stored at –80°C was processed as described previously [21, 23]. The protein concentration of each brain homogenate was determined by the Pierce 660 nm protein assay kit (Pierce Biotechnology, Rockford, IL USA). The tissue homogenates were boiled in Laemmli’s buffer for 5 min and then subjected to 7.5% or 10% SDS-polyacrylamide gel electrophoresis followed by transfer of separated proteins on 0.45-μm Immobilon-P membrane (Millipore, Bedford, MA, USA). The blots were developed using the corresponding primary antibodies (Additional file 1: Table S2). Rabbit monoclonal antiGAPDH (1:2000; Santa Cruz Biotechnology, Santa Cruz, CA, USA) was used for the development of the blots as a loading control. The corresponding horseradish peroxidase-conjugated affinity-pure goat antimouse IgG ((Heavy (H) + Light (L)) and antirabbit IgG (H + L) secondary antibodies were used (1:5000; Jackson ImmunoResearch Laboratories, Inc., West Grove, PA, USA). The blots were visualized using enhanced chemiluminescence (ECL) reagents (Pierce, Rockford, IL, USA). The ECL films of the blots were scanned and then analyzed using Image J (NIH) and Multigauge software (Fuji Photo Film, Co. Ltd). The final quantification of the protein’s band intensity is the result of the subtraction of the background from the original intensity divided by the GAPDH original intensity minus the background. Statistical analysis was conducted using the GraphPad Prism software package, version 5.0 (GraphPad Software Inc., La Jolla, CA, USA), or the Stata 13 statistical package (StataCorp, College Station, TX, USA).

### Statistical analysis

The Stata and GraphPad Prism 5 (GraphPad Software Inc, La Jolla, CA, USA) were used for statistical analysis. Tests of scalar predictors were performed with regression models in Stata 13 (StataCorp, College Station, TX, USA). For multiple group comparisons one-way analysis of variance (ANOVA) followed by Tukey’s post-hoc tests and two-way ANOVA followed by the Bonferroni-adjusted test were used. For all other comparisons (including intergroup comparisons) Student’s *t* test was used. Data are presented as mean ± SD. For all analyses *p* < 0.05 was considered significant.

## Results

### Preventive effect of P021 on synaptic plasticity

#### Synaptic deficit in 3 × Tg-AD mice at 3 months of age before the start of the treatment

**Fig. 2:**
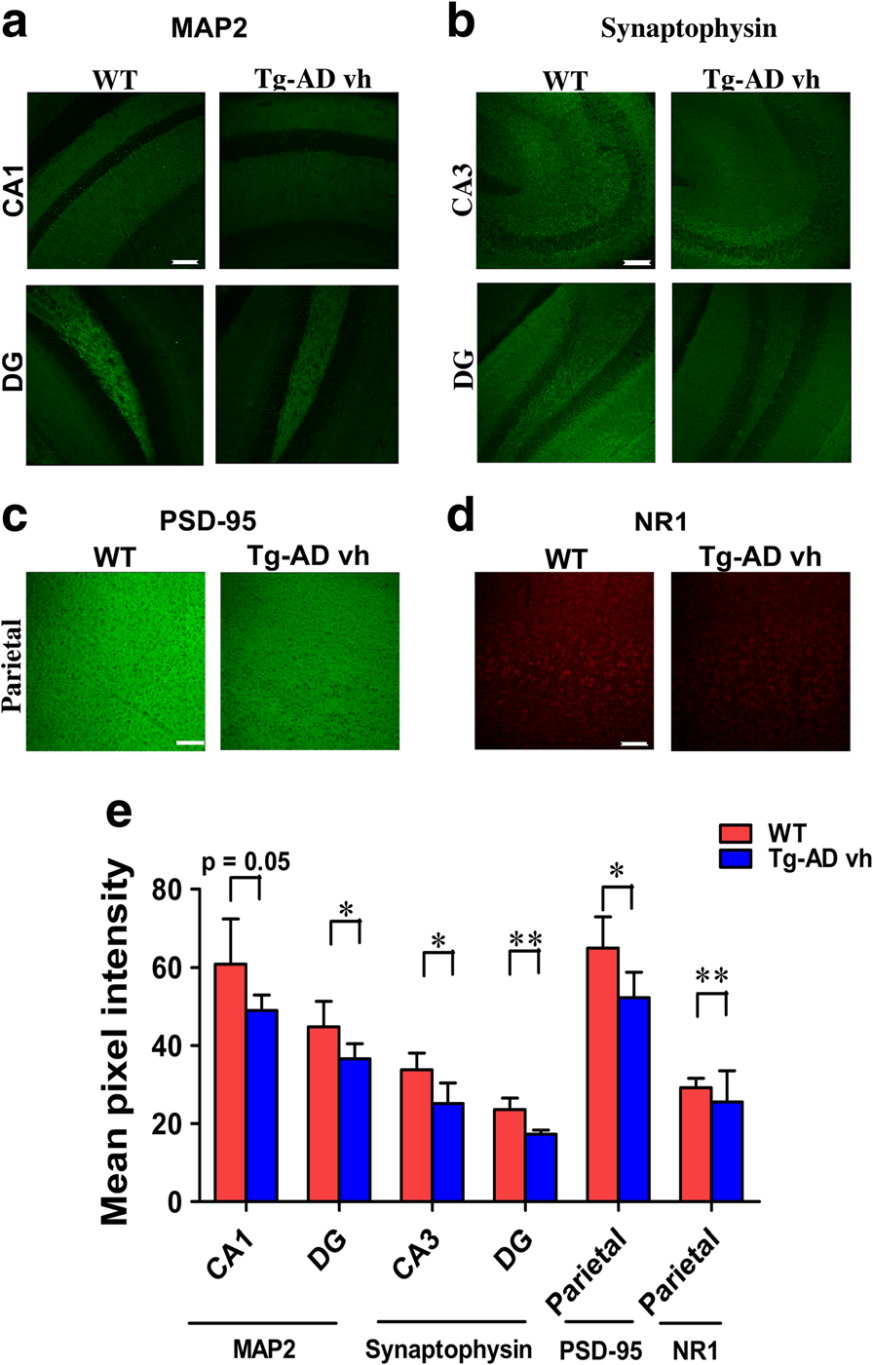
Dendritic and synaptic deficits in 3 × Tg-AD mice at 3 months of age. Expressions of dendritic and synaptic markers were studied immunohistochemically in 3-month-old female 3 × Tg-AD fed a vehicle diet (*Tg-AD vh*) and control wild-type (*WT*) mice. **a** Immunohistochemical staining of MAP2 in the CA1 (stratum radiatum and stratum pyramidae) and dentate gyrus (*DG*; entire area); **b** synaptophysin in CA3 (stratum oriens, stratum radiatum, and stratum pyramidae) and DG; **c** PSD-95 in the parietal cortex (all six layers); **d** NR1 in the parietal cortex; **e** quantification of immunohistochemical staining by mean pixel intensity. At the age of 3 months the 3 × Tg-AD mice show a decrease in the level of expression of several synaptic proteins compared to WT. *p < 0.05, **p < 0.01, by *t* test. *Scale bar*s = 100 μm. WT, *n* = 5; 3 × Tg-AD, *n* = 5. Each maximum projection picture to be analyzed was converted to grayscale and each area to be analyzed was selected, the threshold was adjusted, and the mean pixel intensity of the region of interest was quantified. Quantification is shown as mean ± SD

**Fig. 3:**
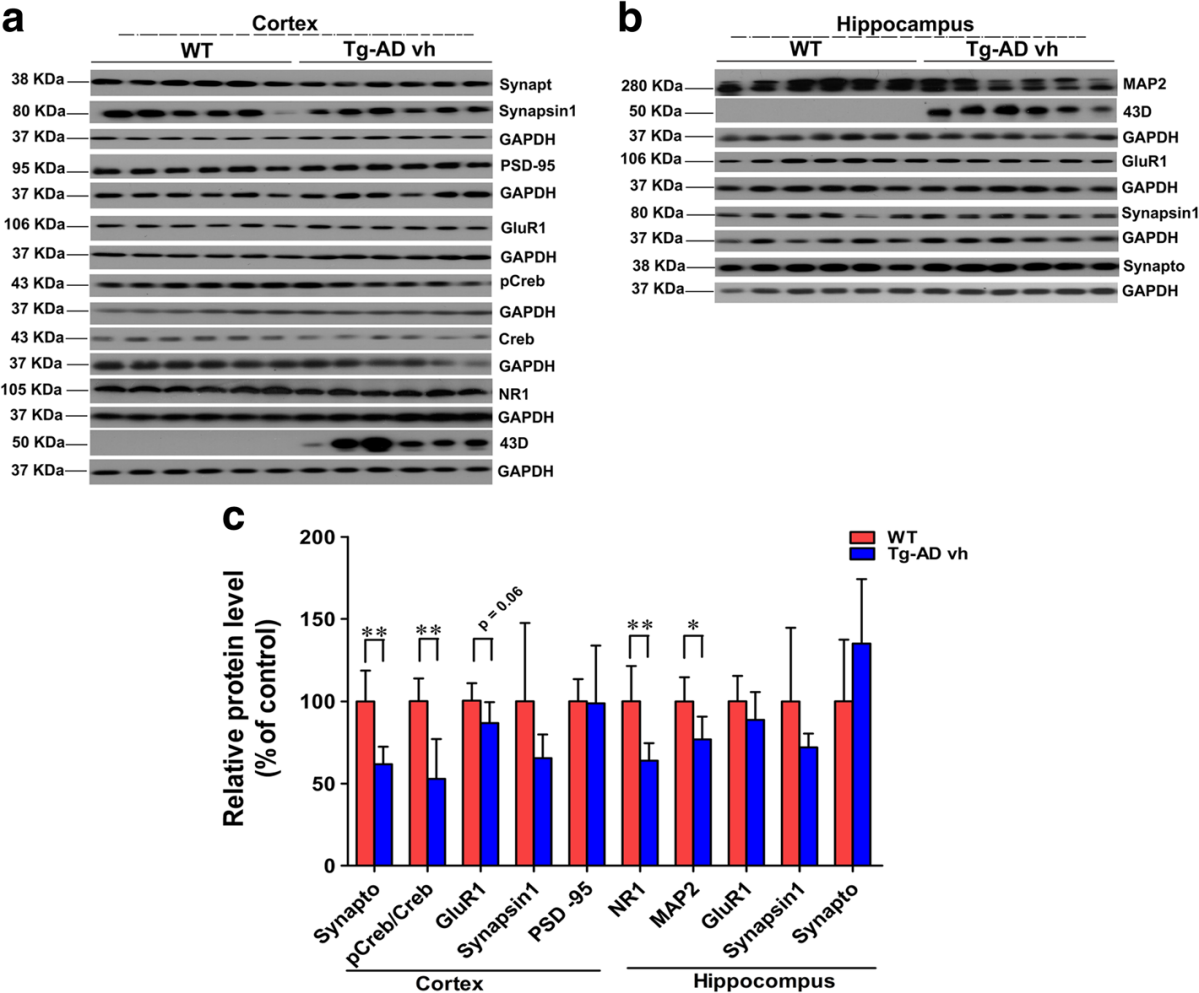
Decreases in levels of cognition-associated proteins in 3-month-old 3 × Tg-AD mice fed a vehicle diet (*Tg-AD vh*). Western blots of **a** the cortex and **b** the hippocampus. **c** Relative quantification of the blots shown in **a** and **b**. All blots were normalized against the loading control GAPDH. The 3 × Tg-AD mice show a decrease in levels of several proteins compared to the control wild-type (*WT*). *p <0.05, **p < 0.01, by *t* test. WT, *n* = 6; 3 × Tg-AD vh, *n* = 6. Quantification is shown as mean ± SD

In a previous study we showed that 3 × Tg-AD mice undergo synaptic compensation and are cognitively impaired at around 3 months [15]. Our aim was to start the treatment with P021 very early in the disease process at around the period of synaptic compensation. We analyzed brain tissues of 3-month-old 3 × Tg-AD mice before the start of the treatment. Along with the synaptic markers we also investigated the expression level of the dendritic marker microtubule-associated protein 2 (MAP2). MAP2 is localized in the somato-dendritic compartment, including the spine, and thus serves as a very useful putative synaptic marker and a marker of differentiated neurons. We studied the expression of MAP2 immunohistochemically and found a trend toward a decrease in the CA1 (Fig. 2a and e; two-tailed Student’s *t* test, *p* = 0.05) and a significant decrease in the DG (Fig. 2a and e; two-tailed Student’s *t* test, *p* = 0.0429). Similarly, Western blot analysis of the hippocampus showed a significant decrease in MAP2 level (Fig. 3b and c; two-tailed Student’s *t* test, *p* = 0.01). Synaptophysin expression was decreased both in the CA3 (Fig. 2b and e; two-tailed Student’s *t* test, *p* = 0.0209) and the DG (Fig. 2b and e; two-tailed Student’s *t* test, *p* = 0.0044). The PSD-95 expression showed a significant difference between the WT and 3 × Tg-AD mice in the parietal cortex (Fig. 2c and e; two-tailed Student’s *t* test, *p* = 0.0395). In the cortex, the level of synaptophysin (Fig. 3a and c; two-tailed Student’s *t* test, *p* = 0.0014) was reduced significantly while that of GluR1 showed a trend towards a decrease in 3 × Tg-AD mice (Fig. 3a and c; two-tailed Student’s *t* test, *p* = 0.06). In the hippocampus, the level and the expression of GluR1 showed no significant difference between the 3 × Tg-AD and the WT mice (Fig. 3b and c). The level of NR1in the cortex and the expression in the parietal cortex decreased significantly in 3 × Tg-AD mice compared to WT (Fig. 2d and e; two-tailed Student’s *t* test, *p* = 0.0014; Fig. 3a and c; two-tailed Student’s *t* test, *p* = 0.004). No significant difference was found in the level of PSD95 between the two groups by Western blot in the cortex and the hippocampus (Fig. 3a and c). The analysis of the level of the ratio of pCREB and CREB in the cortex showed a significant decrease in 3 × Tg-AD compared to WT mice (Fig. 3a and c; two-tailed Student’s *t* test, *p* = 0.002). Human-specific monoclonal antibody 43D to tau was used to confirm the 3 × Tg-AD mice genotype compared to WT (Fig. 3a and b). Collectively, these findings showed dendritic and synaptic deficits and decreased CREB activity in 3 × Tg-AD mice as compared to their WT controls at the age of 3 months.

#### Effect of P021 on synaptic markers after 9 months of treatment in 3 × Tg-AD mice

**Fig. 4:**
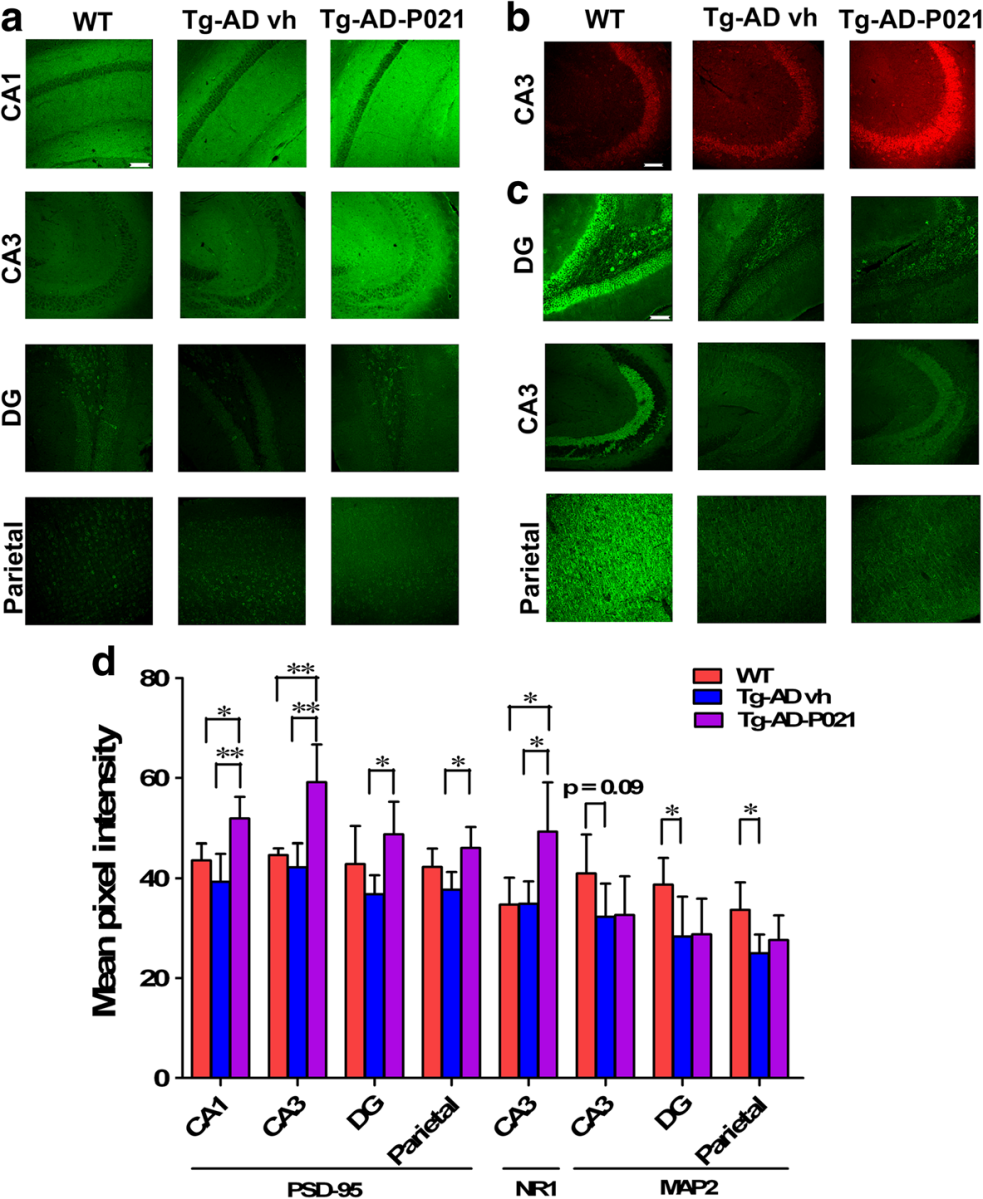
Effect of P021 on reversal of dendritic and synaptic deficits in 3 × Tg-AD mice at 9 months posttreatment. After 9 months of treatment, 3 × Tg-AD mice fed a P021 diet (Tg-AD-P021) and vehicle-treated (*Tg-AD vh*) and wild-type (*WT*) controls mice were euthanized and the expressions of PSD-95, NR1, and MAP2 were studied by immunohistochemical staining. Immunohistochemical staining of: **a** PSD-95 in CA1 (stratum radiatum and stratum pyramidae), CA3 (stratum oriens, stratum radiatum, and stratum pyramidae), dentate gyrus (*DG*; entire area), and the parietal cortex (all six layers); **b** NR1 in CA3; and **c** MAP2 in DG, CA3, and parietal cortex. **d** Quantification of immunohistochemical staining. 3 × Tg-AD-vh showed a decrease in the expression of PSD-95 and NR1, and P021 rescued this deficit in 3 × Tg-AD mice. *p < 0.05, **p < 0.01, by one-way ANOVA post-hoc test. *Scale bar* = 100 μm. WT, *n* = 5; 3 × Tg-AD-vh, *n* = 5; 3 × Tg-AD-P021, *n* = 5. Each maximum projection picture to be analyzed was converted to greyscale and each area to be analyzed was selected, the threshold was adjusted, and the mean pixel intensity of the region of interest was quantified. Quantification is shown as mean ± SD

We investigated the effect of 9 months of treatment with P021 on dendritic and synaptic plasticity both immunohistochemically and biochemically. We found a significant increase in the expression of PSD-95 in 3 × Tg-AD-P021 compared to WT and 3 × Tg-AD-vh mice in the CA1 (Fig. 4a and d; one-way ANOVA, post-hoc *t* test, *p* = 0.0038) and CA3 regions (Fig. 4a and d; one-way ANOVA, post-hoc *t* test, *p* = 0.0010). A significant difference was found between 3 × Tg-AD-vh and 3 × Tg-AD-P021 mice in the DG (Fig. 4a and d; one-way ANOVA, post-hoc *t* test, *p* = 0.0314) and the parietal cortex (Fig. 4a and d; one-way ANOVA, post-hoc *t* test, *p* = 0.0169). Western blot analysis of the hippocampus showed a trend towards a decrease in the level of PDS-95 expression in the 3 × Tg-AD-vh mice compared to WT mice (Additional file 1: Figure S1b and c; two-tailed Student’s *t* test, *p* = 0.07) and a trend towards an increase in 3 × Tg-AD-P021 compared to 3 × Tg-AD-vh mice (Additional file 1: Figure S1b and c; two-tailed Student’s *t* test, *p* = 0.07); more details can be found in the supplemental material. We found that the NR1 expression in the CA3 region increased in 3 × Tg-AD-P021 compared to WT and 3 × Tg-AD-vh mice (Fig. 4b and d; one-way ANOVA, post-hoc *t* test, *p* = 0.0091).

A trend toward a decrease was found in MAP2 expression in the CA3 (Fig. 4c and d; one-way ANOVA, post-hoc *t* test, *p* = 0.09), the DG (Fig. 4c and d; two-tailed Student’s *t* test, *p* = 0.0416) and the parietal cortex (Fig. 4c and d; one-way ANOVA, post-hoc *t* test, *p* = 0.0392) in the 3 × Tg-AD-vh compared to WT mice. However, no apparent effect of P021 to prevent this deficit was detected. In the cortex, the analysis of the level of synapsin 1 showed no difference among the three groups by Western blot (Additional file 1: Figure S1a and c). The level of synaptophysin was significantly decreased in 3 × Tg-AD-vh compared to WT mice (Additional file 1: Figure S1a and c; two-tailed Student’s *t* test, *p* = 0.0339) but there was no significant difference between 3 × Tg-AD-P021 and 3 × Tg-AD-vh mice (Additional file 1: Figure S1a and c). These findings suggested a partial preventive effect of 9 months of treatment with P021 on synaptic deficit in 3 × Tg-AD mice.

#### Effect of P021 on prevention of synaptic deficit after 15 months of treatment

**Fig. 5:**
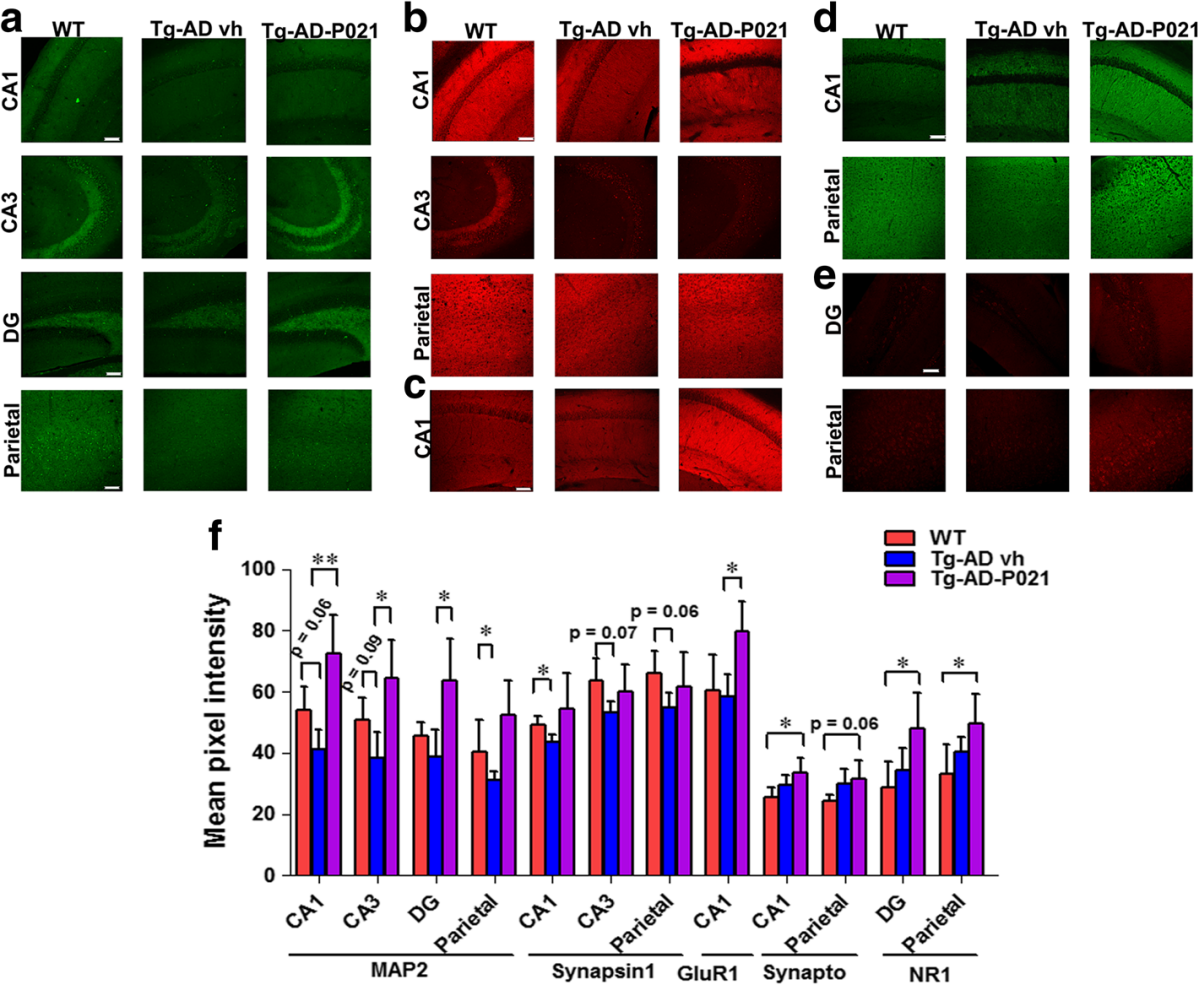
Prevention of dendritic and synaptic deficits by P021 in 3 × Tg-AD mice at 15 months posttreatment. After 15 months, P021- (*Tg-AD-P021*) and vehicle-treated (*Tg-AD vh*) animals were euthanized, and expressions of MAP2, synapsin 1, GluR1, synaptophysin, and NR1 were studied immunohistochemically. **a** Immunohistochemical staining of MAP2 in CA1 (stratum radiatum and stratum pyramidae), CA3 (stratum oriens, stratum radiatum, and stratum pyramidae), dentate gyrus (*DG*; entire area), and parietal cortex (all six layers); **b** synapsin 1 in CA1, CA3, and parietal cortex; **c** GluR1 in CA1; **d** synaptophysin in CA1 and parietal cortex; **e** NR1 in DG and parietal cortex. **f** Quantification of immunohistochemical staining. The 3 × Tg-AD-vh mice show a deficit in the expression of MAP2 and GluR1, and P021 was able to rescue this deficit. *p < 0.05, **p < 0.01, by one-way ANOVA post-hoc test. *Scale bar* = 100 μm. Wild-type (*WT*), *n* = 5; 3 × Tg-AD-vh, *n* = 4; 3 × Tg-AD-P021, *n* = 5. Each maximum projection picture to be analyzed was converted to greyscale and each area to be analyzed was selected, the threshold was adjusted, and the mean pixel intensity of the region of interest was quantified. Quantification is shown as mean ± SD

The immunohistochemical analysis of the expression of MAP2 showed that there was a trend towards a decrease in CA1 in 3 × Tg-AD-vh mice (Fig. 5a and f; two-tailed Student’s *t* test, *p* = 0.06); P021 treatment was detected at 15 months post-treatment to significantly prevent the synaptic deficit in the CA1 (Fig. 5a, f; one-way ANOVA, post-hoc *t* test, *p* = 0.0065). In the CA3 region a trend towards a decrease was detected in 3 × Tg-AD-vh as compared with WT mice (Fig. 5a and f; two-tailed Student’s *t* test, *p* = 0.09) and P021 was found to rescue this deficit (Fig. 5a and f; one-way ANOVA, post-hoc *t* test, *p* = 0.0226). A significant preventive effect of P021 was found in 3 × Tg-AD-P021 compared to 3 × Tg-AD-vh mice in the DG (Fig. 5a and f; one-way ANOVA, post-hoc *t* test, *p* = 0.0239) and the parietal cortex (Fig. 5a and f; one-way ANOVA, post-hoc *t* test, *p* = 0.0491).

The expression of synapsin 1 showed a decrease in the CA1 region (Fig. 5b and f; two-tailed Student’s t test, p = 0.0395), and a trend in the CA3 region (Fig. 5b, f; two-tailed Student’s *t* test, *p* = 0.07) and the parietal cortex (Fig. 5b and f; two-tailed Student’s *t* test, *p* = 0.06) in 3 × Tg-AD-vh compared to WT mice.

The expression of GluR1 showed a significant increase in the 3 × Tg-AD-P021 mice in the CA1 region compared to the 3 × Tg-AD-vh mice (Fig. 5c and f; two-tailed Student’s *t* test, *p* = 0.0365).

The expression of synaptophysin in the CA1 region showed a significant increase in the 3 × Tg-AD-P021 compared to WT mice (Fig. 5d and f; two-tailed Student’s *t* test, *p* = 0.0450) and a trend towards an increase in the parietal cortex in 3 × Tg-AD-P021 compared to WT mice (Fig. 5d and f; two-tailed Student’s *t* test, *p* = 0.0623). The analysis of the expression of NR1 showed that it increased in the DG (Fig. 5e and f; one-way ANOVA, post-hoc *t* test, *p* = 0.0306) and the parietal cortex (Fig. 5e and f; one-way ANOVA, post-hoc *t* test, *p* = 0.0476) of the 3 × Tg-AD-P021 compared to WT mice.

Level of synapsin1 in the cortex showed a preventive effect of P021 (Additional file 1: Figure S2a and c; *p* = 0.0003) in 3 × Tg-AD-P021 mice. Similarly, we found an increase in the level of synaptophysin in the cortex of the 3 × Tg-AD-P021 mice compared to 3 × Tg-AD-vh mice (Additional file 1: Figure S2a and c; two-tailed Student’s *t* test, *p* = 0.0045). The NR1 expression showed a trend towards a positive effect of P021 (Additional file 1: Figure S2a and c). In the hippocampus we found a trend towards a positive effect of P021 on levels of MAP2 and synapsin1 (Additional file 1: Figure S2b and c; two-tailed Student’s *t* test, *p* = 0.05 and *p* = 0.06, respectively). Collectively, these findings revealed that 15 months of treatment with P021 can prevent dendritic and synaptic deficits in 3 × Tg-AD mice.

#### Effect of P021 on synaptic and dendritic deficits after 18 months of treatment

**Fig. 6:**
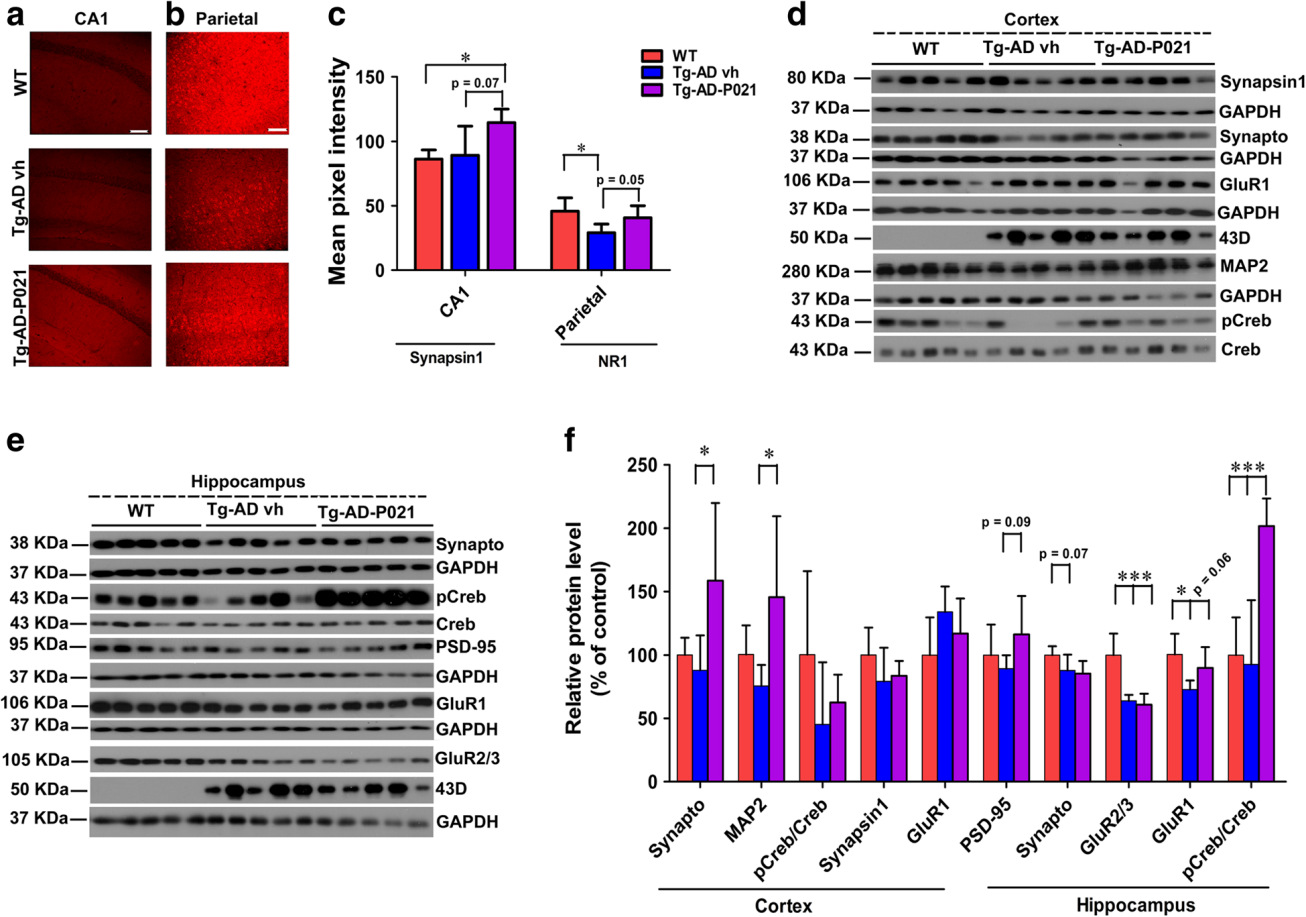
Prevention of dendritic and synaptic deficits and rescue of CREB activity loss by P021 in 3 × Tg-AD mice at 18 month posttreatment. After 18 months, P021- (*Tg-AD-P021*) and vehicle-treated (*Tg-AD vh*) mice were euthanized and expressions and/or levels of synapsin 1, NR1, synaptophysin, MAP2, phosphor- (*pCREB*) and total CREB, synapsin 1, GluR1, and PSD 95 were studied immunohistochemically or by Western blot. **a** Immunohistochemical analysis of synapsin 1 expression in CA1 (stratum radiatum and stratum pyramidae) and **b** NR1in parietal cortex (all six layers). **c** Quantification of immunohistochemical staining shown in **a** and **b. d** Western blots of synaptic proteins in the whole cortex and **e** the whole hippocampus. **f** Relative quantification of the blots. All blots were normalized against the loading control GAPDH. The level and the expression of several synaptic markers were deficient in the 3 × Tg-AD-vh mice and P021 was able to rescue this deficit. *p < 0.05, ***p < 0.001, by one-way ANOVA post-hoc test. *Scale bar* = 100 μm. Wild-type (*WT*), *n* = 5; Tg-AD-vh, *n* = 5; Tg-AD-P021, *n* = 5. Each maximum projection picture to be analyzed was converted to greyscale and each area to be analyzed was selected, the threshold was adjusted, and the mean pixel intensity of the region of interest was quantified. Quantification is shown as mean ± SD

We found a significant increase in synapsin 1 expression in the CA1 region in 3 × Tg-AD-P021 compared to WT mice (Fig. 6a and c; two-tailed Student’s *t* test, *p* = 0.0384) and a trend towards an increase compared to 3 × Tg-AD-vh mice (Fig. 6a and c; two-tailed Student’s *t* test, *p* = 0.0791). The expression of NR1 in the parietal cortex was decreased in the 3 × Tg-AD-vh compared to WT mice (Fig. 6b and c; two-tailed Student’s *t* test, *p* = 0.0305) and there was a trend towards an increase in 3 × Tg-AD-P021 compared to 3 × Tg-AD-vh mice (Fig. 6b and c; two-tailed Student’s *t* test, *p* = 0.05).

We found that P021 treatment significantly increased the level of synaptophysin in the cortex but not the hippocampus in the 3 × Tg-AD mice (Fig. 6d and f; one-way ANOVA, post-hoc *t* test, *p* = 0.03). The MAP2 level was significantly increased in the cortex of 3 × Tg-AD-P021 compared to 3 × Tg-AD-vh animals (Fig. 6d and f; two-tailed Student’s *t* test, *p* = 0.045). The level of PSD-95 in the hippocampus showed a trend towards an increase in 3 × Tg-AD-P021 compared to 3 × Tg-AD-vh mice (Fig. 6e and f; two-tailed Student’s *t* test, *p* = 0.09). GluR1 expression was significantly decreased in the hippocampus of 3 × Tg-AD-vh mice (Fig. 6e and f; one-way ANOVA, post-hoc *t* test, *p* = 0.02) and P021 treatment showed a trend in rescuing this (Fig. 6e and f; two-tailed Student’s *t* test, *p* = 0.06). The level of the ratio of pCreb and Creb showed a significant increase in the 3 × Tg-AD-P021 group compared to the other two groups (Fig. 6e and f; one-way ANOVA, post-hoc *t* test, *p* = 0.0007). The GluR2/3 level showed a significant increase in the WT group compared to the other two groups (Fig. 6e and f; one-way ANOVA, post-hoc *t* test, *p* = 0.0002). Collectively, these findings revealed that chronic treatment with P021 can prevent dendritic and synaptic deficits and loss of CREB activity in 3 × Tg-AD mice.

### Effect of P021 on neurogenesis

**Fig. 7:**
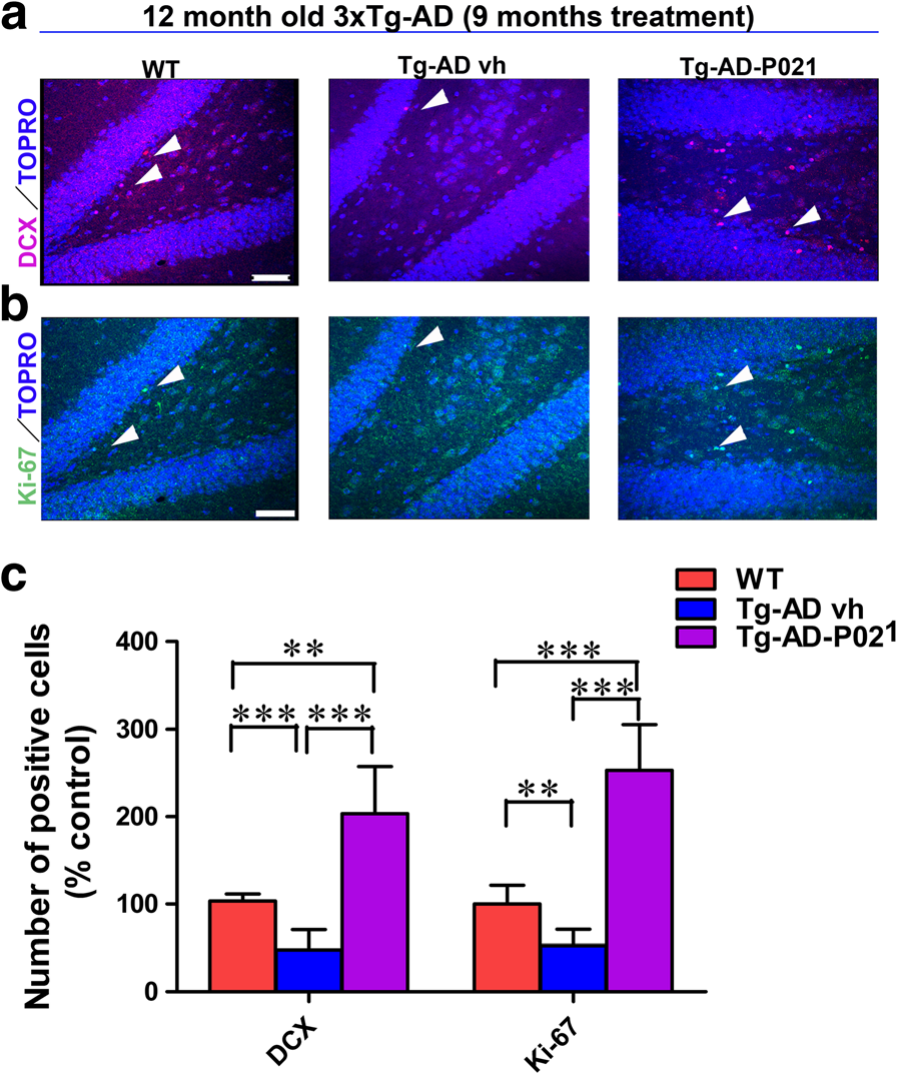
Rescue of DG neurogenesis deficit after 9 month posttreatment with P021 in 3 × Tg-AD mice. After 9 months of treatment, P021- (*Tg-AD-P021*) and vehicle-treated (*Tg-AD vh*) mice were killed and their brains were analyzed for neurogenesis by doublecortin (*DCX*) and Ki67 immunohistochemical stainings. **a** DCX and TOPRO staining in wild-type (*WT*), 3 × Tg-AD-vh, and Tg-AD-P021mice; **b** Ki-67 and TOPRO staining in WT, 3 × Tg-AD-vh, and 3 × Tg-AD-P021 mice. **c** Densiometric quantification of the number of DCX-positive and Ki-67-positive cells. Labeled cells in the granule cell layer and the hilus of the DG of the hippocampus were manually quantified using a 40× oil objective of a Nikon 90i fluorescent microscope. The number of DCX-positive cells and Ki-67-positive cells was analyzed as the percent of control. Neurogenesis was deficient in the 3 × Tg-AD-vh and P021 was able to boost it in the P021-treated mice. *Arrowheads* indicate positive cells. **p < 0.01, ***p < 0.001, by one-way ANOVA post-hoc test. *Scale bar* = 50 μm. Quantification is shown as mean ± SD

The effect of P021 on neurogenesis was investigated at 9 months posttreatment by immunohistochemical Ki-67 staining, a cell proliferation marker that measures early stages of the neurogenesis, and by DCX staining, a marker for immature newly born neurons. As expected, we found that the number of DCX-positive cells was significantly reduced in the 3 × Tg-AD-vh compared to WT mice (Fig. 7a and c; two-tailed Student’s *t* test, *p* = 0.0009) and P021 rescued this deficit (Fig. 7a and c; one-way ANOVA, post-hoc *t* test, *p* < 0.0001). Remarkably, the level of neurogenesis was rescued to more than the level in the WT group with P021 (Fig. 7a and c; one-way ANOVA, post-hoc *t* test, *p* < 0.001). Similarly, a decrease in the number of Ki-67-positive cells was observed in the 3 × Tg-AD-vh mice compared to WT mice (Fig. 7b and c; two-tailed Student’s t test, *p* = 0.0057) and this deficit was rescued with the P021 treatment (Fig. 7b and c; *p* < 0.0001).

### Effect of P021 on rescuing cognitive deficit

#### Treatment with P021 can rescue reference memory deficit

**Fig. 8:**
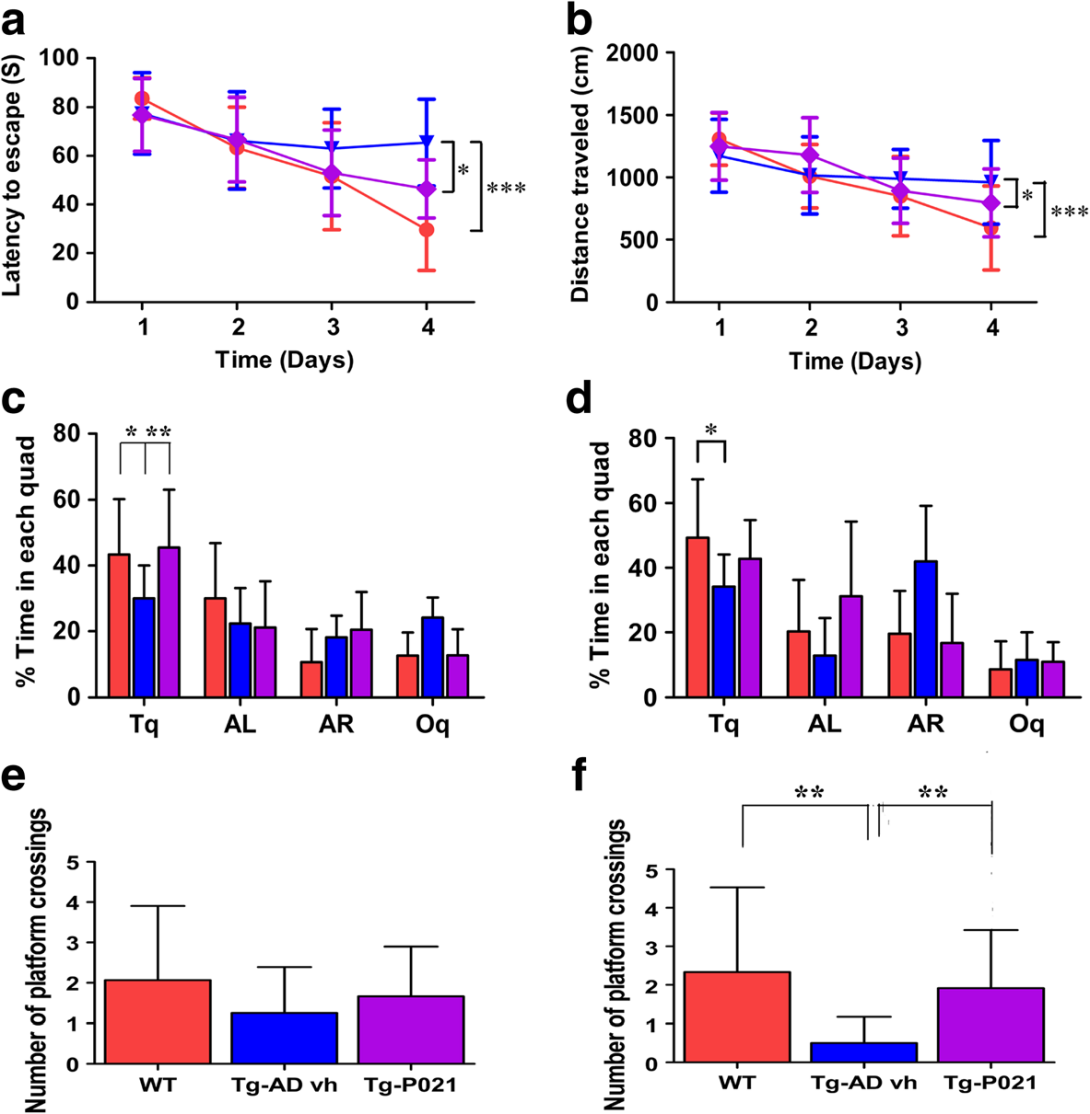
Reversal of reference memory impairment at 9 months posttreatment with P021 in 3 × Tg-AD mice. A set of animals separate from those employed for immunohistochemical and biochemical studies (Figs. 2, 3, 4, 5, 6 and 7) were treated with P021 diet (*Tg-AD-P021*) or vehicle diet (*Tg-AD vh*) and tested for behavioral changes to study the effect of the treatment on reversal of cognitive impairment in 3 × Tg-AD mice by the Morris Water Maze task. **a** Latency to escape; **b** distance traveled; **c** percent of time in target quadrant in probe trial 1 (PT1); **d** in PT2; **e** number of platform crossings in PT1 and **f** and in PT2. Animals were trained for four trials/day for 4 days. PT1 was conducted at 24 h after the last training session; PT2 was conducted at 20 days from PT1. The 3 × Tg-AD-vh mice were impaired in the Morris Water Maze task and P021 was able to prevent this deficit. *p < 0.05, **p < 0.01, ***p < 0.001. Wild-type (*WT*), *n* = 20; 3 × Tg-AD-vh, *n* = 15; 3 × Tg-AD-P021, *n* = 14. Data are shown as mean ± SD. *AL* adjacent left, *AR* adjacent right; *Oq* opposite quadrant, *Tq* target quadrant

The 3 × Tg-AD mice are known to be cognitively impaired very early in the disease process at around 2–3 months of age with regards to spatial reference memory [15, 24] and episodic memory [25]. Since the Morris Water Maze task measures spatial reference memory that is mainly hippocampal-dependent, which is affected early in AD patients, we investigated the effect of P021 treatment on prevention of this impairment in 3 × Tg-AD mice. The Morris Water Maze task was conducted after 9 months of treatment with P021, when the animals were aged 12 months. During the task animals went through four training trials per day for 4 days for a total of 16 trials. There was no difference in the swim speed during the 4 training days (data not shown). The regression analysis of the latency to escape to the hidden platform showed that the 3 × Tg-AD-vh mice took longer than the WT mice (Fig. 8b; *p* < 0.001) and the 3 × Tg-AD-P021 mice (Fig. 8b; *z* = 4.78, *p* = 0.031). The analysis of distance by day showed that 3 × Tg-AD-vh mice learned slower than WT mice (Fig. 8; *p* <0.0001) and 3 × Tg-AD-P021 animals (Fig. 8; *p* = 0.039), and that they traveled farther to find the escape platform. These data showed that the 3 × Tg-AD-vh mice were impaired and that P021 was able to reverse this impairment.

To evaluate retention memory in the 3 × Tg-AD mice, two probe trials (PT) were conducted. The percent of time spent in the target quadrant and the number of crossings of the previous place of the platform were determined to evaluate the performance of the animals. During PT1, the 3 × Tg-AD-vh mice spent significantly less time in the target quadrant compared to WT mice (Fig. 8d; post-hoc Bonferroni-adjusted test, *p <* 0.05) and P021 treatment was able to reverse this impairment (Fig. 8d; post-hoc Bonferroni-adjusted test, *p <* 0.01). A similar trend was seen in the number of visits to the previous place of the platform but there was no significant difference between the three groups (Fig. 8f). During PT2, the analysis of the percent of time in the target quadrant showed that the 3 × Tg-AD-vh mice spent less time compared to the WT mice (Fig. 8e; *p <* 0.05). Furthermore, the analysis of the number of crossings of the previous place of the platform showed that the 3 × Tg-AD-vh mice had made fewer visits compared to the WT mice (Fig. 8g; Student’s *t* test, *p =*0.0094) and that P021 reversed this impairment (Fig.8g; Student’s *t* test, *p* = 0.007). Collectively, these results suggest that memory retention was compromised in 3 × Tg-AD-vh mice and that the 9-month treatment with P021 rescued this impairment.

#### Treatment with P021 can rescue neophobia and short-term spatial memory impairment

##### Neophobia

**Fig. 9:**
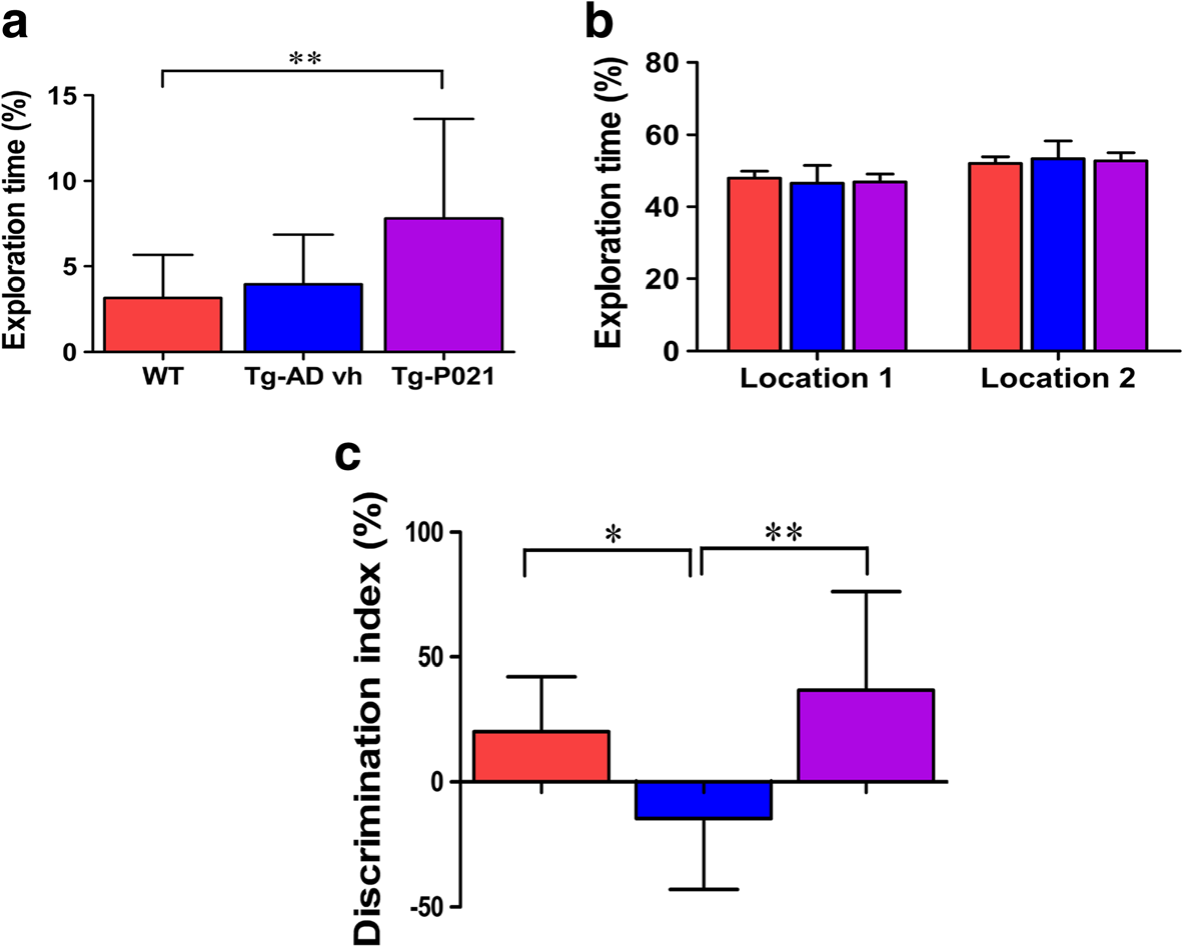
Reversal of neophobia and short-term spatial memory impairment with P021 in 3 × Tg-AD (*Tg-P021*) mice. **a** Neophobia test results. **b** Sample phase and **c** test phase of the novel object location task. In the neophobia test mice were exposed to an unfamiliar object in the center of the open-field apparatus for 5 min and the percent of exploration time was recorded. The novel object location test comprised a habituation phase, a sample phase, and a test phase. During habituation, the animals were trained a total of six sessions, two sessions/day, and each session lasted 10 min within 2 h of intertrial interval. The sample phase and the test phase were conducted 24 h after the last habituation session. The test phase was 1 h after the sample phase. The vehicle-treated mice (*Tg-AD vh*) were impaired in the object location task and P021 was able to prevent this deficit. *p < 0.05, **p < 0.01. Wild-type (*WT*), *n* = 20; 3 × Tg-AD-vh, *n* = 15; 3 × Tg-AD-P021, *n* = 14. Data are shown as mean ± SD

The neophobia test is employed to measure the rodent’s fear of discovering novelty, in this case a novel object. Neophobia is controlled by the amygdala and the ventral region of the hippocampus [26]. In this test, each animal was exposed to an unfamiliar object in the center of an open-field apparatus and left to explore it for 5 min. It was conducted during the first habituation session of the novel object location test. Neophobic animals visit the center of the arena less and explore the novel object less. The statistical analysis of the percent of time spent in the center exploring the novel object showed that both the WT and 3 × Tg-AD-vh mice spent similar time exploring the new object, while the P021-treated mice spent significantly more time exploring the novel object compared to the other two groups (Fig. 9a; one-way ANOVA post-hoc test, *p =* 0.0086).

##### Short-term spatial memory impairment

The novel object location in the open field is employed to measure a hippocampal function since this brain structure is known to be implicated in associating objects with locations [27]. The test was conducted 24 h after the last habituation session and it comprised a sample phase and a test phase. The analysis of the data from the sample phase showed that, as expected, there was no difference in exploring the two identical locations between the three groups of mice (Fig. 9b). However, the test phase showed that the 3 × Tg-AD-vh mice spent more time investigating the familiar location than the novel one, while the WT and 3 × Tg-AD-P021 groups spent more time exploring the novel location than the familiar one (Fig. 9c; ANOVA post-hoc test, *p =* 0.0072). Together, these data showed that the 3 × Tg-AD-vh mice at the age of 18 months had impairment in short-term spatial memory and that the approximate 15 months of treatment with P021 reversed this impairment.

## Discussion

The AD brain undergoes a slow chronic progressive neurodegeneration in which the hippocampus is most affected. At early stages of the disease the brain attempts to repair itself by promoting dentate gyrus neurogenesis and synaptogenesis but, probably because of insufficient appropriate neurotrophic support, this attempt does not succeed beyond a limited time and the degree of dementia keeps advancing [1–7]. Employing 3 × Tg-AD mice as a model of this phenomenon [15], in the present study we found that by providing neurotrophic support with P021 we can rescue synaptic deficit and reverse cognitive impairment. The use of transgenic mouse models has helped understand the molecular mechanisms of AD and has facilitated preclinical studies that could lead to development of potential AD drugs. The 3 × Tg-AD mouse model has proven to be a biologically relevant model to AD pathology. As in AD, the 3 × Tg-AD mice develop deficiency in episodic [24] and spatial reference memories as early as 2–3 months of age that deteriorate with the severity of the disease with advancing age [23]. Also as in AD, in 3 × Tg-AD mice Aβ and tau pathologies and neurodegeneration develop in an age-dependent progressive manner and with similar brain distribution of the lesions. Furthermore, in a separate study, we found that, reminiscent to AD, the 3 × Tg-AD mice show synaptic compensation [15].

In the present study we found that at 3 months of age, before the start of P021 treatment, there were dendritic and synaptic deficits as seen by the decrease in the levels of MAP2, synaptophysin, PSD-95, and NR1 in 3 × Tg-AD mice. These findings are consistent with a reduction in synaptophysin expression during early AD reported previously [28–30]. Dendritic spine loss in 5- to 6-month-old 3 × Tg-AD mice under short-term modern life-like stress was also reported previously [31]. At 9 months posttreatment we found P021 was able to rescue synaptic deficit as seen immunohistochemically by PSD-95 and NR1 staining. At 15 months posttreatment P021 was also able to rescue both dendritic and synaptic deficits as seen by an increase in levels and expressions of MAP2, synaptophysin, synapsin 1, GluR1, and NR1 both immunohistochemically and biochemically. Similar data were reported previously in studies where 6- to 11-month-old animals were treated with P021 or its parent molecule, Peptide 6 [8, 13, 20, 21, 23, 32, 33]. In the present study, at 18 months posttreatment P021 prevented synaptic deficit as shown by immunohistochemical stainings and Western blot. Thus, collectively, the present study shows that early chronic treatment with P021 can be effective even until later age in rescuing neuronal plasticity deficits in 3 × Tg-AD mice.

The level of neurogenesis was investigated by the quantification of the number of DCX-positive and Ki-67-positive cells in the DG area at 9 months posttreatment with P021. The results showed that neurogenesis was defective in 3 × Tg-AD-vh mice and that the treatment with P021 was able to rescue this deficit. This neurogenic effect of P021 could be through the inhibition of the leukemia inhibitory factor (LIF) signaling pathway or the increase in the BDNF expression as described by us previously [21]. These results are in agreement with those shown previously when P021 was injected subcutaneously into normal adult C57Bl6/J mice [13] or by oral gavage to a rat model of cognitive aging [12]. Similar data were also reported with 7,8-dihydroxyflavone (7,8-DHF) which is a neurotrophic compound that acts as a BDNF agonist [34, 35]. The rescue of cognitive impairment by P021 treatment found in the present study is consistent with similar beneficial effects shown previously with 7,8-DHF [36, 37] and other BDNF agonist compounds [38, 39].

The results of this study emphasize the importance of early intervention in human clinical trials, especially during the period where the brain still has the capacity to repair itself and before extensive synaptic and neuronal loss. This way, a healthy neuronal network can be preserved and can prevent or slow down the progression of AD. Since sporadic AD is not detected before the appearance of the first clinical symptoms, the strategy that we are proposing can benefit from the number of reliable biomarkers such as cerebrospinal fluid (CSF) levels of tau and Aβ and neuroimaging, especially with tau and Aβ tracers developed recently in the field. For instance, biomarkers can be used to define people that are at risk for developing AD and then the prevention treatment with P021 can be initiated. In the present preclinical study of almost 2 years, P021 did not show any severe side effects or safety issues. However, more extensive pharmacokinetic and pharmacodynamic studies will be required before its safety and efficacy in AD patients can be tested.

Although the results of the present study are encouraging they have to be interpreted with caution. While the 3 × Tg-AD model shows synaptic and cognitive deficit very early in the disease process and mimics most of the neuropathological features of AD and has proven to be a suitable mouse model to help test different drugs for AD in preclinical studies, it does not recapitulate the mechanism and full comprehensive neuropathology of human AD. The 3 × Tg-AD mouse is an overexpression model which obviously does not represent the human disease mechanism. Another limit of this study is that we used only female mice. The decision to not use both genders in this study was made because there are disparities between males and females in the consistency and the aggressiveness of the pathology. Several laboratories independently reported that only female 3 × Tg-AD animals show consistent and aggressive pathology [17–19]. The 3 × Tg-AD mouse model was generated by the co-injection of two mutated transgenes, human βAPP_Swe_ and tau P301L, in PS1_M146v_ knock-in embryos. This is a homozygous mouse line and these animals and their genetically matched 129/Sv × C57BL/6 control WT mice are independently bred. While a lack of littermate control is not ideal for comparing the differences between 3 × Tg-AD and the WT animals, the comparison of P021-treated with the vehicle-treated 3 × Tg-AD mice employed in the present study clearly showed the therapeutic beneficial effect of P021 on both neurobiology and neurobehavior of the AD mouse model. In the present study we employed naive animals for neurobiological studies. Our previous studies showed that P021 rescues Aβ and tau pathologies in both behaviorally tested (Morris water maze and novel object discrimination tasks) and untested 3 × Tg-AD mice, and that the mice kept on a vehicle diet have higher Aβ and tau pathology burden than those treated with P021; P021 decreased both pathologies markedly and rescued cognitive deficit in 3 × Tg-AD mice [21, 40].

The sporadic form of AD which represents over 95–99% of the cases is not caused by any mutation, which makes this mouse model representing more the familial form than the sporadic form of the disease. The insertion of the transgenes into the genome may knockout other genes creating artifacts that are not present in human AD. However, this is an inherent problem for all the transgenic mice available in the field. What explains our choice of the 3 × Tg-AD mouse in this study is that it shows the synaptic compensation phenomenon [15] that is the main focus of the therapeutic intervention in the present study.

## Conclusions

In conclusion, the prevention of dendritic and synaptic deficits and reversal of cognitive impairment by chronic treatment started during the period of synaptic compensation with a neurotrophic compound in an AD mouse model shows the therapeutic potential of this strategy. Unlike neurotrophic factors which are blood–brain barrier impermeable and cause serious side effects such as anorexia, severe cramps, and muscle pain by CNTF, treatment with P021 for around 18 months did not show any weight loss, tumors, or signs of pain in 3 × Tg-AD mice.

## Additional file

Additional file 1:
**Table S1.** Number of animals used. **Table S2.** Antibodies used. **Figure S1.** Rescue of synaptic deficit with P021 in 3 × Tg-AD mice at 9 months posttreatment. Western blots: a) cortex; b) hippocampus; and c) relative quantification of the blots. All blots were normalized against the loading control GAPDH. One-way ANOVA post-hoc test. **p* < 0.05. WT, *n* = 5; Tg-AD-vh, *n* = 5; Tg-AD-P021, *n* = 5. Quantification data are shown as mean ± SD. **Figure S2.** Preventive effect of P021 on levels of dendritic and synaptic markers in 3 × Tg-AD mice at 15 months posttreatment. Western blots: a) cortex; b) hippocampus; and c) relative quantification of the blots. The 3 × Tg-AD-vh show a decrease in the level of synaptic markers and P021 was able to rescue this deficit. All blots were normalized against the loading control GAPDH. One-way ANOVA post-hoc test. ***p* < 0.01. WT, *n* = 5; Tg-AD-vh, *n* = 3; Tg-AD-P021, *n* = 5. Quantification data are shown as mean ± SD. (DOCX 5530 kb)

## Abbreviations

3 × Tg-AD mouse: Triple transgenic mouse model of Alzheimer’s disease
7,8-DHF: 7,8- Dihydroxyflavone
AD: Alzheimer’s disease
ANOVA: Analysis of variance
Aβ: Amyloid beta
BDNF: Brain-derived neurotrophic factor
CA1: Cornu ammonis 1
CA3: Cornu ammonis 3
CNTF: Ciliary neurotrophic factor
CREB: cAMP response-element binding protein
CSF: Cerebrospinal fluid
DCX: Doublecortin
DG: Dentate gyrus
ECL: Enhanced chemiluminescence
FGF: Fibroblast growth factor
GluR1: Glutamate receptor 1
GluR2/3: Glutamate receptor 2/3
LIF: Leukemia inhibitory factor
MAP2: Microtubule-associated protein 2
NFT: Neurofibrillary tangles
NR1: NMDA receptor 1
P021: Peptide 021
PBS: Phosphate-buffered saline
PS1: Presenilin 1
PSD-95: Postsynaptic density-95
PT1: Probe trial 1
PT2: Probe trial 2
WT: Wild type
βAPP_Swe_: Beta amyloid precursor protein with Swedish mutation
