# Supplementary material for: Prevention of dendritic and synaptic deficits and cognitive impairment with a neurotrophic compound
Source: Alzheimers Res Ther. 2017 Jun 27;9:45. doi: 10.1186/s13195-017-0273-7 (PMC5488423; doi:10.1186/s13195-017-0273-7)
Supplement: Additional file 1: — Table S1. Number of animals used. Table S2. Antibodies used. Figure S1. Rescue of synaptic deficit with P021 in 3 × Tg-AD mice at 9 months posttreatment. Western blots: a) cortex; b) hippocampus; and c) relative quantification of the blots. All blots were normalized against the loading control GAPDH. One-way ANOVA post-hoc test. *p < 0.05. WT, n = 5; Tg-AD-vh, n = 5; Tg-AD-P021, n = 5. Quantification data are shown as mean ± SD. Figure S2. Preventive effect of P021 on levels of dendritic and synaptic markers in 3 × Tg-AD mice at 15 months posttreatment. Western blots: a) cortex; b) hippocampus; and c) relative quantification of the blots. The 3 × Tg-AD-vh show a decrease in the level of synaptic markers and P021 was able to rescue this deficit. All blots were normalized against the loading control GAPDH. One-way ANOVA post-hoc test. **p < 0.01. WT, n = 5; Tg-AD-vh, n = 3; Tg-AD-P021, n = 5. Quantification data are shown as mean ± SD. (DOCX 5530 kb) [file 13195_2017_273_MOESM1_ESM.docx]

**Supplemental material Alzheimer’s Research & Therapy**

**Prevention of dendritic and synaptic deficits and cognitive impairment with a neurotrophic compound**

Narjes Baazaoui ^a,b^ and Khalid Iqbal^a*^

^a^ Department of Neurochemistry, Inge Grundke-Iqbal Research Floor

New York State Institute for Basic Research in Developmental Disabilities

1050 Forest Hill Road, Staten Island, NY 10314, USA.

^b^ Graduate Program in Biology (Neuroscience), College of Staten Island (CSI)

City University of New York (CUNY) Graduate Center

365 5th Ave, New York, NY 10016, USA.

**Corresponding Author:**

Khalid Iqbal, Ph.D.

Chairman, Department of Neurochemistry

New York State Institute for Basic Research in Developmental Disabilities,

1050 Forest Hill Road, Staten Island, NY 10314, USA

Phone: 1-718 494-5259; Fax: 1-718-494-1080

E-mail:  [khalid.iqbal.ibr@gmail.com](mailto:khalid.iqbal.ibr@gmail.com)

### **Table S1. Number of animals used**

| **Animals used** |  |
| --- | --- |
| **Immunohistochemistry and Western blots**  21 WT; 20 3xTg-AD-vh; 18 3xTg-AD-P021  3 months (WT, n = 6; 3xTg-AD, n =6)  12 months (WT, n = 5; 3xTg-AD-vh, n = 5; 3xTg-AD-P021, n = 5)  18 months (WT, n = 5; 3xTg-AD-vh, n = 4; 3xTg-AD-P021, n = 5)  21 months (WT, n = 5; 3xTg-AD-vh, n = 5; 3xTg-AD-P021, n = 6) | **Behavioral studies**  20 WT; 15 3xTg-AD-vh; 14 3xTg-AD-P021 |

**Table S2: Antibodies used**

| **Antibody** | **Dilution** | **company** | **Catalog #** | **IHC** | **WB** |
| --- | --- | --- | --- | --- | --- |
| GluR1 | 1:300 IHC  1:1000 WB | EMD Millipore | AB1504 | yes | yes |
| Synaptophysin | 1:500 IHC  1:3000 WB | Millipore | 5258 | yes | yes |
| MAP2 | 1:1000 IHC  1:4000 WB | Covance | SMI-52R | yes | yes |
| PSD95 | 1: 1000 WB  1:100 IHC | Sigma  Cell signaling | **P-246 (IHC)**  3450S (WB) | yes | yes |
| 43D | 1:1000 | BioLegend | 816601 | no | yes |
| NR1 | 1:200 IHC  1:500 WB | ABCAM | ab17345 | yes | yes |
| GluR 2/3 | 1:2000 | ABCAM | 52896 | no | yes |
| DCX | 1:50 IHC | Santa Cruz | sc-8066 | yes | no |
| Ki-67 | 1:50 IHC | Santa Cruz | sc-7846 | yes | no |
| Synapsin-1 | 1:2000 IHC  1:40000 WB | Stress Gene | VAP-SV060 | yes | yes |
| GAPDH | 1:2000 WB | ABCAM | Ab 2485 | no | yes |
| CREB | 1:1000 WB | Cell signaling technology | 9197 | no | yes |
| p-CREB | 1:1000 WB | Cell signaling technology | 9198 | no | yes |





**Fig. S1: Rescue of synaptic deficit with P021 in 3xTg-AD mice at 9 months post-treatment.** Western blots, a) cortex; b) hippocampus and c) relative quantification of the blots. All blots were normalized against the loading control GAPDH. One Way ANOVA post hoc test. *p<0.05. (WT, n = 5; Tg-AD-vh, n = 5; Tg-AD-P021, n = 5). Quantification data are shown as mean ± SD.


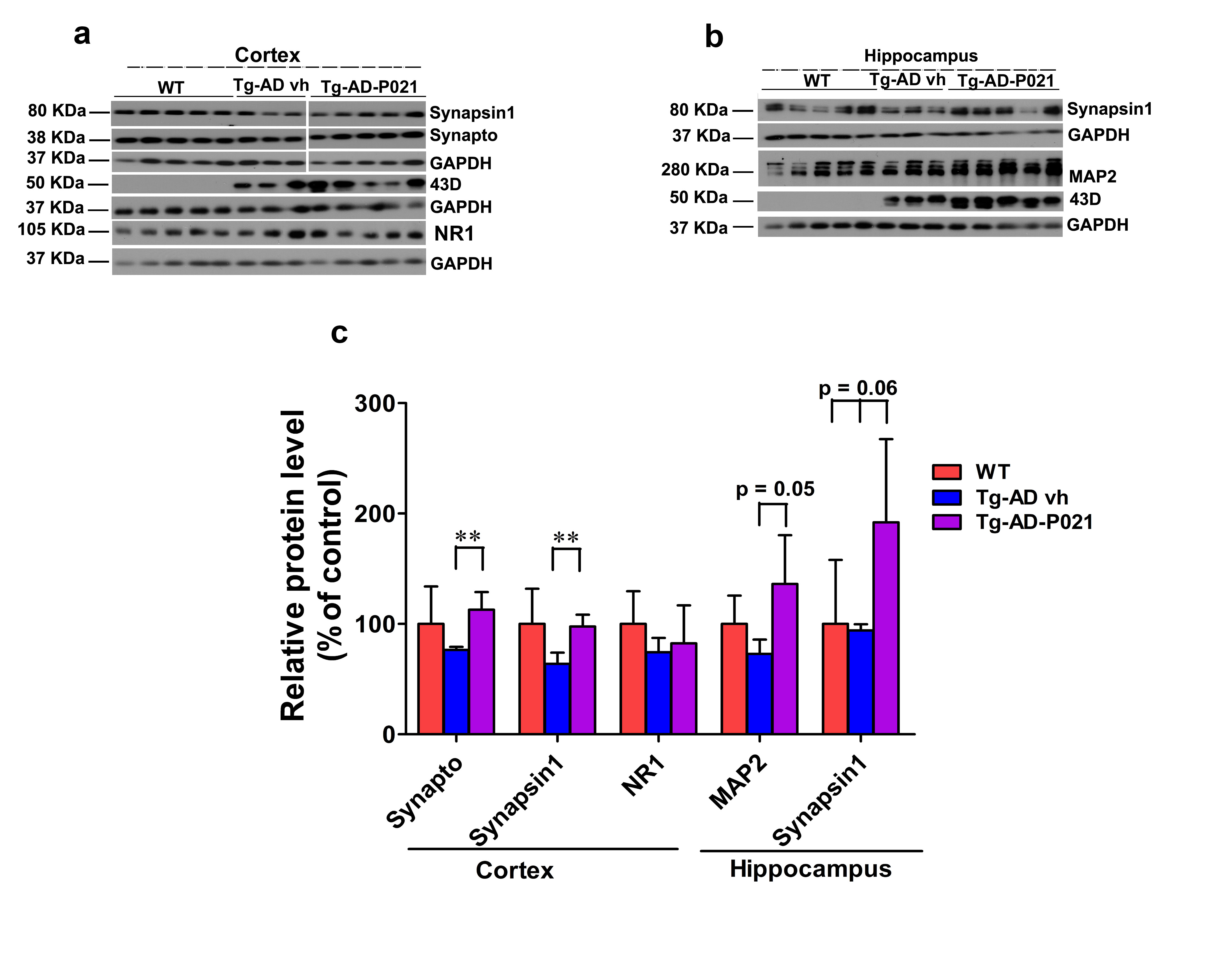


## Fig. S2: Preventive effect of P021 on levels of dendritic and synaptic markers in 3xTg-AD mice at 15 months post-treatment. Western blots a) cortex; b) hippocampus and c) relative quantification of the blots. The 3xTg-AD-Vh show decrease in the level of synaptic markers and P021 was able to rescue this deficit. All blots were normalized against the loading control GAPDH. One Way ANOVA post hoc test. **p<0.01. (WT, n = 5; Tg-AD-vh, n = 3; Tg-AD-P021, n = 5). Quantification data are shown as mean ± SD.
